# The Impact of 3D Stacking and Technology Scaling on the Power and Area of Stereo Matching Processors

**DOI:** 10.3390/s17020426

**Published:** 2017-02-22

**Authors:** Seung-Ho Ok, Yong-Hwan Lee, Jae Hoon Shim, Sung Kyu Lim, Byungin Moon

**Affiliations:** 1 Samsung Electronics, Hwaseong-si, Gyeonggi-do 18448, Korea; seungho.ok@samsung.com; 2 School of Electronic Engineering, Kumoh National Institute of Technology, Gumi 39177, Korea; yhlee@kumoh.ac.kr; 3 School of Electronics Engineering, Kyungpook National University, Daegu 41566, Korea; jhshim@knu.ac.kr; 4 School of Electrical and Computer Engineering, Georgia Institute of Technology, Atlanta, GA 30332, USA; limsk@ece.gatech.edu

**Keywords:** through-silicon via, stereo matching processor, technology scaling, low-power

## Abstract

Recently, stereo matching processors have been adopted in real-time embedded systems such as intelligent robots and autonomous vehicles, which require minimal hardware resources and low power consumption. Meanwhile, thanks to the through-silicon via (TSV), three-dimensional (3D) stacking technology has emerged as a practical solution to achieving the desired requirements of a high-performance circuit. In this paper, we present the benefits of 3D stacking and process technology scaling on stereo matching processors. We implemented 2-tier 3D-stacked stereo matching processors with GlobalFoundries 130-nm and Nangate 45-nm process design kits and compare them with their two-dimensional (2D) counterparts to identify comprehensive design benefits. In addition, we examine the findings from various analyses to identify the power benefits of 3D-stacked integrated circuit (IC) and device technology advancements. From experiments, we observe that the proposed 3D-stacked ICs, compared to their 2D IC counterparts, obtain 43% area, 13% power, and 14% wire length reductions. In addition, we present a logic partitioning method suitable for a pipeline-based hardware architecture that minimizes the use of TSVs.

## 1. Introduction

Various types of sensors that provide three-dimensional (3D) depth information include stereo cameras, radar, and time-of-flight cameras [1,2]. Recently, to obtain a dense depth map from a pair of stereo images, stereo matching processors have been adopted in real-time embedded systems such as intelligent robots and autonomous vehicles [3,4,5]. Important requirements for stereo matching processors are a high frame rate with minimal hardware resources and low power consumption [6]. In this paper, to achieve these requirements in the design of stereo matching processors, we adopt a TSV-based 3D stacking technology that has emerged as a practical solution for hardware miniaturization and low-power circuits. The stereo matching processor requires a wide bandwidth because of the memory-intensive nature of stereo image processing. Therefore, stereo matching processors are promising candidates for fully exploiting the benefits of 3D stacking technology.

Several related studies have examined the benefits of 3D stacking by comparing 3D integrated circuits (ICs) with their two-dimensional (2D) IC counterparts. Ouyang et al. described the design of 3D-stacked arithmetic units [7]. Thorolfsson et al. presented a 3D-stacked fast Fourier transform (FFT) processor composed of memory on logic and compared it with its 2D IC counterparts [8]. For a pair comparison, they used the same synthesis output, but they did not compare power consumption under the same clock frequency. Neela et al. implemented a 3D-stacked single precision floating-point unit [9], but they did not use the same netlist output for a pair comparison of 2D and 3D IC designs. Kim et al. showed the benefits of the TSV-based 3D integration of a many-core processor and memory stacking [10], and Zhang et al. showed the feasibility of 3D-stacked dynamic random access memory (DRAM) for multimedia applications [11]. A reconfigurable memory on a logic tier using face-to-face bonding, a 3D-stacked digital signal processor, and a 3D ternary content-addressable memory were demonstrated in [12,13,14]. Table 1 provides a summary of the related work on TSV-based 3D-stacked IC design.

In contrast to the goals of the work mentioned above [7,8,9,10,11,12,13,14], one of the primary goals of this research is to analyze the influence of reduced wire length that results from vertical stacking on power consumption by comparing 3D ICs with their 2D IC counterparts. In addition, we expand our previous work [15] to investigate the impact of technology process scaling (130 nm down to 45 nm) and TSV scaling (2.2 μm down to 0.8 μm) on the power and area benefits of 3D stacking. We design our 3D-stacked stereo matching processors using GlobalFoundries 130 nm and Nangate 45 nm process design kits (PDKs) and compare them with their 2D IC counterparts regarding power consumption and wire length. For a fair comparison of 3D and 2D ICs, we use the same target clock period and logic synthesis output during implementation. In this work, we also propose a pipeline-based partitioning scheme that reduces TSV use and then compare its performance with that of a conventional partitioning method. Our study is based on graphic database system II (GDSII)-level layouts, on the sign-off performance, and on a power analysis for a highly accurate assessment of the issues.

Overall, we summarize our contributions as follows: (1) We show the impact of 3D stacking on power consumption based on a practical implementation of 3D ICs; (2) to show the impact of partitioning on 3D ICs, we present two types of partitioning methods: the proposed pipeline-level partitioning and conventional macro-level partitioning; (3) we provide design considerations for low-power 3D IC design from a comprehensive analysis and implementation results; and (4) we study the impact of both device and TSV scaling on the power and area benefits of 3D ICs. This paper is divided into the following sections: Section 2 describes the stereo matching algorithm and its hardware architecture, and Section 3 discusses the design environments, design flow, and analysis flow. Section 4 compares the overall layout, presents the results of the detailed power analysis, and discusses the impact of switching activity, all of which are discussed in Section 5.

## 2. Stereo Matching Processor

### 2.1. Matching Algorithm

**Figure 1:**
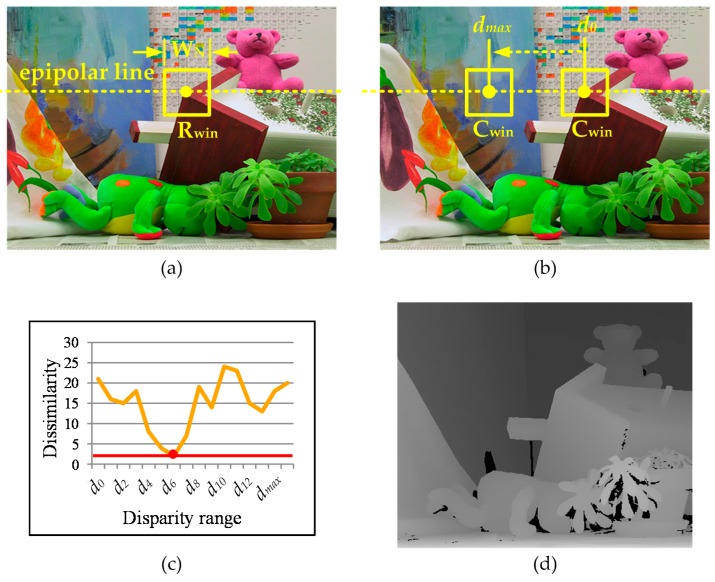
(**a**) Left image (R_win_: reference window); (**b**) right image (d_x_: disparity range, C_win_: candidate window); (**c**) dissimilarity between R_win_ and C_win_; and (**d**) a depth map.

Stereo matching involves the identification of corresponding pixels in a pair of stereo images and the extraction of three-dimensional information by computing the disparity between corresponding pixels by triangulation [1]. It often requires a huge amount of signal processing and a wide memory bandwidth for real-time stereo matching. Over the last decade, a large variety of stereo matching algorithms that can be grouped into local and global matching algorithms have been developed [2]. Local algorithms are faster than global algorithms, but the latter tend to be more accurate. Thus, the global algorithm is often implemented on a special platform such as graphic processing units because of its substantial computational overhead. Unlike the global algorithm, the local algorithm performs matching operations on the one-dimensional epipolar line by comparing the correlations of windows in a given search range, shown in Figure 1. As a result of this computational approach, the computational overhead of the local algorithm is less than that of the global algorithm, so the local algorithm is frequently adopted in embedded applications. We designed stereo matching processors based on a window-based local matching algorithm that requires a fixed size of window because this algorithm is more straightforward in a pipelined hardware architecture. We use 44 SRAM macros so that the window-based operations can handle the requirements of a wide bandwidth and the real-time processing capability of the matching algorithm.

**Figure 2:**
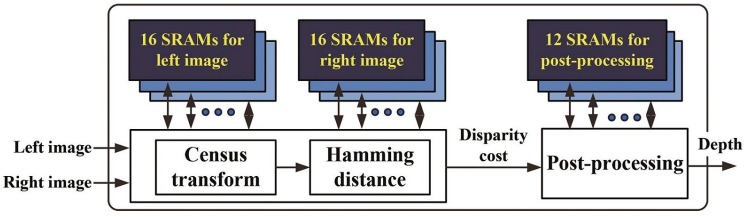
Flow diagram of the stereo matching processor.

The most basic step in the matching algorithm is the computation of the matching cost, which presents the similarity between the reference and candidate windows. It can be computed by several methods such as the sum of absolute differences and census transforms [16,17]. Several comprehensive studies have compared the matching accuracy of various types of matching cost computation methods, and several studies that have found that rank and census transforms are inherently robust to radiometric distortions of images have observed that the census transform outperforms other window-based stereo matching methods [18,19]. Thus, we adopt the census transform, which presents the characteristic feature of a window as a sequence of bit streams [20,21]. Figure 2 presents a flow diagram of the stereo matching processor, which performs an 11 × 11 window-based census transform using stereo images to find corresponding pixels in a pair of stereo images and computes the matching cost of the window based on the Hamming distance. After the post-processing of the matching results, the stereo matching processor generates a dense depth map.

### 2.2. Hardware Architecture

**Figure 3:**
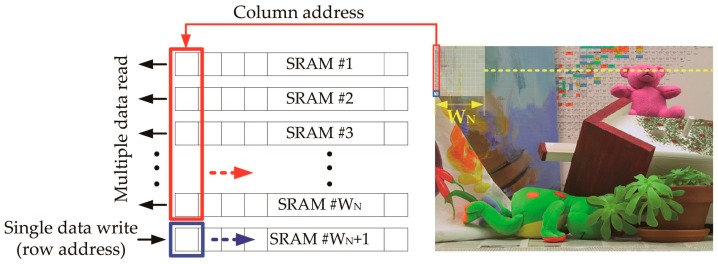
Illustration of the multiple-read, single-write operation of the stereo matching algorithm.

To run the matching algorithm using a pair of stereo images, the window-based stereo matching algorithm requires sufficient memory. Thus, the primary goal of the stereo matching architecture is to efficiently buffer both the left and right images on the memory so that the stereo matching processor can generate a depth map in real time. To fulfill this requirement and to deal with the requirement of a wide bandwidth, we used small, highly partitioned SRAMs and conducted a window-based operation using a finite number of them. Figure 3 illustrates how the window is generated with the finite number of SRAMs and then propagated horizontally. A pair of stereo images are consecutively acquired by a stereo camera, stored in the SRAMs in rows, and processed in columns for window-based matching. As a result, during each cycle, this architecture can concurrently perform multiple reads and a single write operation. Thus, the stereo matching processor can handle the requirements of a wide bandwidth.

**Figure 4:**
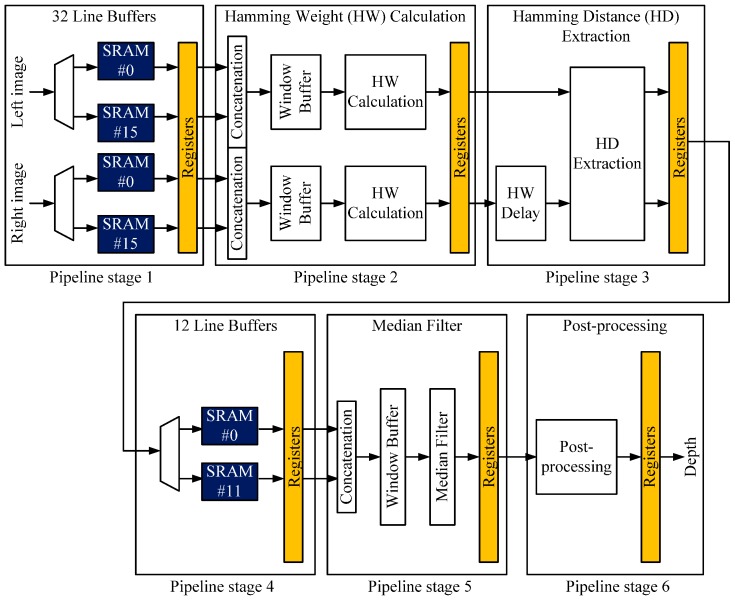
Pipelined hardware architecture of our stereo matching processor.

As shown in Figure 3, the size of memory is determined primarily by the width of the image and the height of the window. The former equals the depth of each SRAM and the latter the number of SRAMs. As the width of the image increases, therefore, the size of the SRAM increases; as the size of the window increases, the required number of SRAMs increases accordingly. However, a large number of interconnections between the SRAMs and logic cells will cause performance degradation resulting from high routing congestion and the longer wire lengths in 2D ICs. We used an eight-bit, gray-level 752 × 480 image and a 15 × 15 window for the stereo matching and an 11 × 11 window for the post-processing. We chose these window sizes because of their high degree of matching accuracy [22]. Figure 4 shows the fully pipelined hardware architecture of the stereo matching processor, and Table 2 summarizes the features of the stereo matching processors.

## 3. Design and Analysis Flow

### 3.1. Design Environments

**Figure 5:**
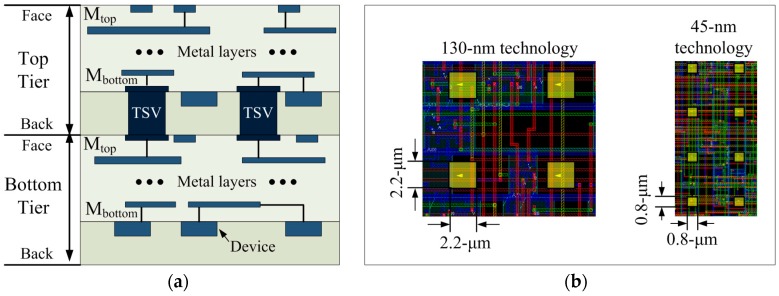
Via-first bonding technology used in this paper: (**a**) Side view of via-first TSVs; and (**b**) top-down view of TSVs.

We designed our 2D and 3D ICs using GlobalFoundries 130-nm and Nangate 45-nm process design kits (PDKs). To generate 44 single-port SRAM macros composed of six-transistor bit cells, we used an ARM memory compiler (ARM Inc., San Jose, CA, USA). We chose this commercial technology setting because it was successfully used in the development of a 3D IC built with GlobalFoundries 130-nm process technology and Tezzaron’s TSV-based vertical stacking technology [10]. We connected two tiers of 3D ICs using via-first TSVs with the face-to-back bonding style for 3D integration, shown in Figure 5. The diameters of the TSVs were 2.2 μm for the 130-nm and 0.8 μm for the 45-nm designs. Because the capacitance and resistance of TSVs are not negligible in timing and power analyses, we used 10 fF and 2 fF for the capacitance of the TSV and 50 mΩ and 10 mΩ for the resistance of the TSV, respectively, during the timing and power analyses of our 130-nm and 45-nm designs. We used simulated values for the capacitance and resistance of TSVs.

### 3.2. Design Flow

**Figure 6:**
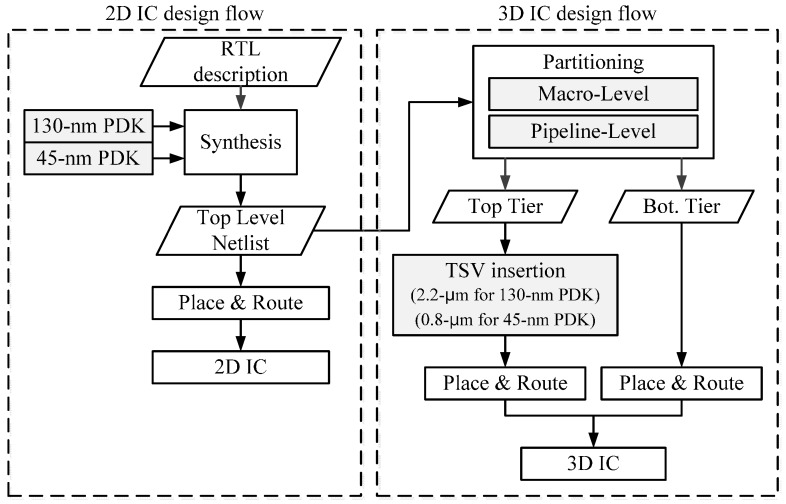
2D and 3D IC design flow.

Figure 6 presents the design flow for the 2D and 3D IC designs. From the given register transfer level (RTL) description of the stereo matching processor written in Verilog hardware description language (HDL), we used a conventional design flow for the 2D IC design. We generated top-level synthesized netlists of the 130-nm and 45-nm designs using Synopsys’s Design Compiler and two types of PDKs. For a fair comparison of the 2D and 3D ICs, we used the same synthesized netlist for both. For the 2D IC layout, we performed floor planning, placement, clock-tree synthesis, routing, and timing optimization using Cadence Encounter. Table 3 summarizes the results of the synthesis.

For the primary step of the 3D IC design, we used two methods of partitioning. In one method, we separated the gates of the functional modules and the SRAM macros in the top-level netlist, assigned them to the top and bottom tiers, respectively, and then determined the required number of TSVs needed for interconnecting each tier. In the other method, we partitioned the top-level synthesized netlist in a pipeline-level style using “group” and “ungroup” commands in the Synopsys Design Compiler; we then extracted the partitioned netlist for each tier and inserted the TSVs into the netlist. The “ungroup” and “group” commands are used to remove the hierarchy of the top-level synthesized netlist and to create two partitioned netlists for two tiers of 3D IC design, respectively. We placed the TSVs prior to gate placement and did the layout separately for each tier in the same way that we performed the conventional 2D IC layout. Table 4 presents a comparison between the TSV usage of the proposed pipeline-level and conventional macro-level partitioning methods. For the macro-level partitioned 3D IC design, we uniformly placed 425 signal TSVs according to the location of the input and output of each SRAM macro. In the case of the pipeline-level partitioned 3D IC design, we simply placed 221 signal TSVs in the center area of the top tier because most of them, which came from logic cells on the top tier, connected to memory macros on the bottom tier. We did not optimize the locations of the TSVs, which was beyond the scope of this study.

#### 3.2.1. Macro-Level Partitioning (MP) Method

**Figure 7:**
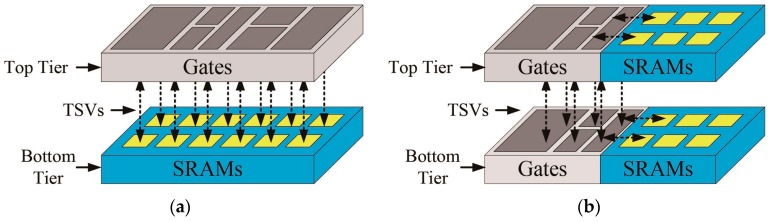
(**a**) The conventional macro-level partitioning method; and (**b**) the proposed pipeline-level partitioning method.

We divided the top-level netlist into logic gates and memory macros, shown in Figure 7a. As the gates are placed vertically over the memory macros, this partitioning method minimizes the wire lengths between the logic and memory macros, thus maximizing the benefits of the 3D ICs. Because all TSVs and die sizes are proportional to the number the macros, as the number of macros increases, the number of TSVs and sizes of dies also increase. In this case, if the total area of the macros is not proportional to the total area of the logic cells, maintaining a balance between the utilization ratios of each tier is difficult. Moreover, the larger number of TSVs increases the overhead of the silicon area, resulting in routing congestion.

#### 3.2.2. Pipeline-Level Partitioning (PP) Method

**Figure 8:**
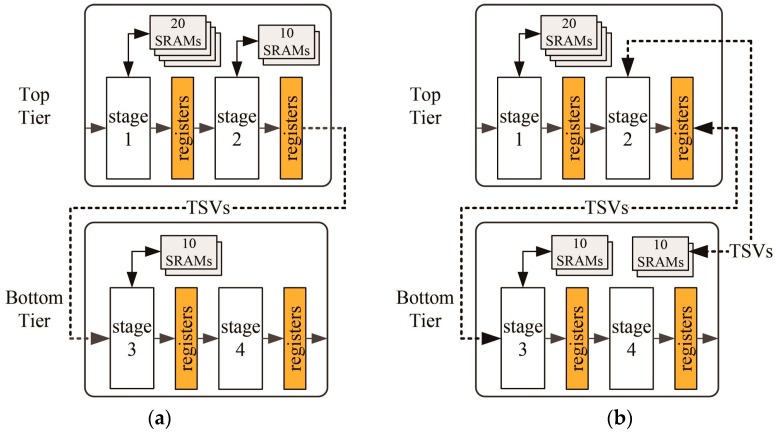
An illustration of the proposed pipeline-level partitioning method: (**a**) Split the pipeline stages into two tiers, and (**b**) adjust the number of SRAMs in each tier.

The purpose of this partitioning method is to minimize the number of TSVs and to balance the die sizes of the tiers with minimal effort in a pipelined hardware architecture. In this partitioning method, we adhere to the basic concept of pipelining (in which memory is dedicated to its own pipeline stage, shown in Figure 4) by simply splitting the pipeline stages into two groups and assigning the first three pipeline stages (1, 2 and 3) to the top tier and the remaining pipeline stages (4, 5 and 6) to the bottom tier. In this case, the number of TSVs is determined by the number of signals between the two groups. However, simply dividing the pipeline stages into two groups does not guarantee a balance of die sizes of the tiers. Therefore, we balanced them by moving the memory macros from the top to the bottom tier. In this case, the number of TSVs increased as the number of adjusted memory macros increased. Figure 8 illustrates the two steps of the pipeline-level partitioning method. In this example, we assumed that the silicon areas of each stage were identical.

#### 3.2.3. Comparison of the Partitioning Methods

For the 3D IC design, we used both the conventional macro-level and proposed pipeline-level partitioning methods. The 3D IC with the macro-level partitioning method (3D-MP), which consists of a logic tier (top tier) and a memory macro tier (bottom tier), minimizes wire lengths between the logic and memory macros, shown in Figure 7a. For the 3D-MP design, we uniformly placed 425 signal TSVs on the top tier according to the location of the input and output ports of each memory macro. For the 3D IC design with the pipeline-level partitioning method (3D-PP), we split the pipeline stages to minimize the number of TSVs and then balanced the cell area of each tier by adjusting the number of memory macros of each tier. We assigned the first three pipeline stages (1, 2 and 3) of Figure 4 to the top tier, and the remaining pipeline stages (4, 5 and 6) to the bottom tier. In this case, before adjusting the number of memory macros of each tier, we assigned 67.5% of the cell area to the top tier and 32.5% to the bottom tier. Thus, with the same footprint size, the top tier will suffer more from routing congestion. From the cell area report, shown in Table 5, we learned that each SRAM macro occupies about 1.4% of the cell area, so to balance the cell area of each tier, we moved 12 SRAM macros from the top to the bottom tier. For the 3D-PP design, we placed the cells in pipeline stages 3 and 4 in the central part of the top and bottom tiers and connected them using TSVs located in the central part of the layout mainly because the cells in pipelines 1 and 6 are placed on the boundary of the die for the pad connection. In addition, since it was not the goal of this study to find the optimal locations of TSVs for 3D ICs, we placed the TSVs in the center area of the chip for the 3D-PP design.

### 3.3. Timing and Power Analysis Flow

**Figure 9:**
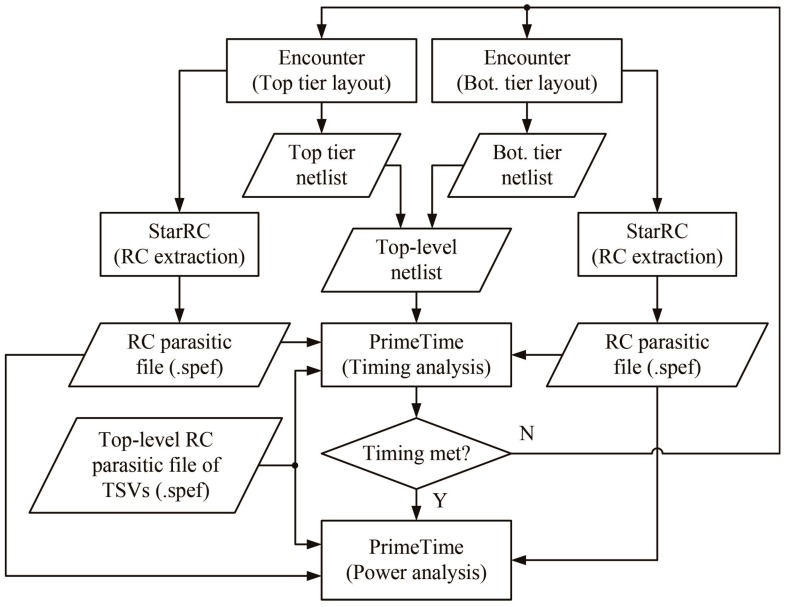
Overall flow of the power and timing analyses for a 3D IC.

We conducted a static timing analysis (STA) using Synopsys PrimeTime with the layout netlist and an RC parasitic file that contained the resistance and capacitance values for all of the nets. Then, if the timing was met, we performed a power analysis using PrimeTime. Figure 9 presents the flow of the power and timing analyses. We performed the timing and power analyses using PrimeTime with the layout netlist and the RC parasitic file of each tier. Although existing commercial tools can perform a timing and power analyses for 2D IC design, they cannot handle 3D IC designs. Thus, for the 3D IC analysis, we created a top-level netlist by combining the netlist of the tiers and a top-level RC parasitic file for the TSVs. Then, we merged three parasitic files (two from the two dies and one from the TSVs) into one and used it to perform 3D timing analysis. We also used the combined parasitic files to obtain timing constraints at the die boundary and performed timing optimization. If the timing was met, we conducted a power analysis. Our power comparisons were done in iso-performance. We used the same target clock period during the layout and timing optimization for both the 2D and 3D designs. We used 3.2 ns for the 130-nm and 1.8 ns for the 45-nm 2D and 3D ICs. The clock-tree synthesis for the 3D IC was difficult because no commercial EDA tools are able to fully handle clock trees for 3D ICs. Thus, we treated each tier as if it had its own clock-tree network and then performed clock-tree synthesis separately. Then, we directly connected the clock sources of the top and bottom tiers through a TSV.

## 4. Experimental Results

### 4.1. Overall Layout Comparisons

**Figure 10:**
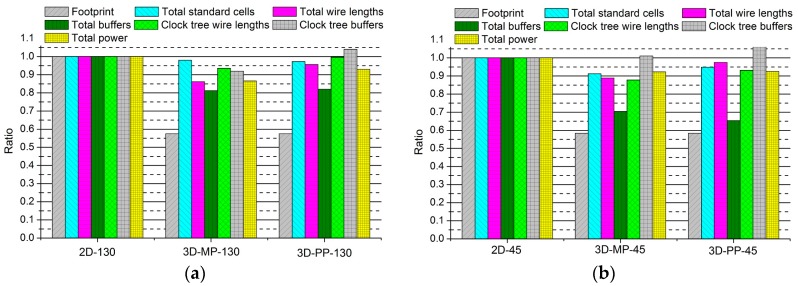
Comparisons between the normalized designs of 2D and 3D ICs: (**a**) 2D and 3D ICs designed in 130-nm process technology; and (**b**) 2D and 3D ICs designed in 45-nm process technology.

**Figure 11:**
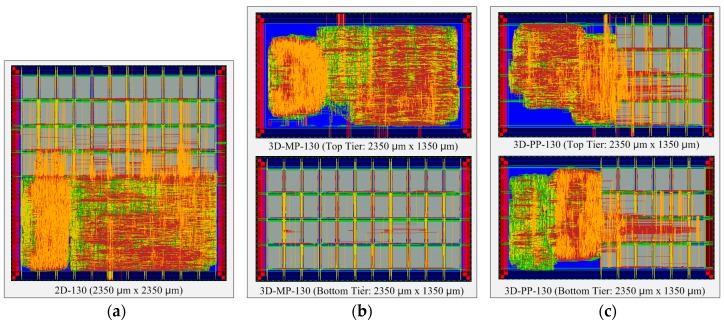
Layout snapshots of 2D and 3D ICs designed in 130-nm process technology: (**a**) 2D IC (2D-130); (**b**) the top and bottom tiers of a 3D IC using macro-level partitioning (3D-MP-130); and (**c**) the top and bottom tiers of a 3D IC using pipeline-level partitioning (3D-PP-130).

**Figure 12:**
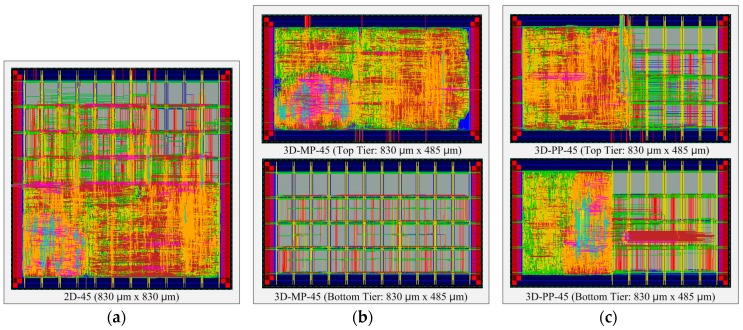
Layout snapshots of 2D and 3D ICs designed in 45-nm process technology: (**a**) 2D IC (2D-45); (**b**) the top and bottom tiers of a 3D IC using macro-level partitioning (3D-MP-45); and (**c**) the top and bottom tiers of a 3D IC using pipeline-level partitioning (3D-PP-45).

Figure 10 presents the comparisons between the normalized design quality of the 2D and 3D ICs, whose layout snapshots, designed in 130-nm and 45-nm technologies, are shown in Figure 11 and Figure 12, respectively, and whose overall layout results are summarized in Table 6 and Table 7. First of all, we observe that the 3D ICs deliver significant performance improvements over the 2D ICs in their overall metrics: footprint, power, and wire length. The chip footprints are as much as 43% smaller than those of the 2D ICs in both the 130-nm and 45-nm process technology-based 3D ICs. In addition, in the case of the 130-nm process technology-based 3D ICs (3D-MP-130 and 3D-PP-130), the total wire lengths are 14% and 4%, respectively, shorter than those of the 2D IC (2D-130) because of vertical stacking and smaller footprints. We also observe that the total number of buffers of the 3D-MP-130 and 3D-PP-130 are nearly 19% and 18% less than that of the 2D-130, mainly because of shortened wire lengths. As a result, 3D-MP-130 and 3D-PP-130 consume 13% and 7% less power, respectively, than 2D-130. Similarly, the total wire lengths of the 45-nm process technology-based 3D ICs (3D-MP-45 and 3D-PP-45) are 11% and 3% shorter than that of the 2D IC (2D-45); the numbers of buffers are 30% and 35% less than that of 2D-45; and the power consumption of 3D-MP-45 and 3D-PP-45 is 8% and 7% less than that of 2D-45, respectively.

In contrast to the results of the comparison of footprints, wire lengths, buffers, and power, the results of 3D stacking from the clock-tree analysis did not exhibit any benefits. In the case of the 3D-PP-130, although the wire length of the clock tree was 7% shorter than that of the 2D-130, its number of clock-tree buffers was 4% higher than that of the 2D IC. Similarly, the number of clock-tree buffers of 3D-MP-45 and 3D-PP-45 were 1% and 10% higher, respectively, than that of 2D-45. One explanation for this finding is that we performed clock-tree synthesis separately for the top and bottom tiers and then directly connected the clock source of the bottom to that of the top tier through a TSV. In this case, we used existing commercial electronic design automation (EDA) tools to perform the clock-tree synthesis without any awareness of the other clock tree. Thus, the clock tree of the 3D IC was not well optimized. As 3D-MP-130 and 3D-MP-45, however, did not have a large clock-tree network on the bottom tier, they suffered less from the clock-tree optimization problem.

Compared with 3D-PP, the 3D-MP uses a larger number of TSVs but outperforms 3D-PP, particularly regarding the wire length and the number of clock tree buffers. This finding could result from several factors: (1) the placement of TSVs in 3D-PP may not be optimal; or (2) the clock-tree network of 3D-PP is not optimized, so it has more clock buffers than that of 3D-MP. From these observations, we learn that the optimal placement of TSVs and the synthesis of the 3D clock tree play important roles in 3D IC design.

### 4.2. Detailed Power Analysis

**Figure 13:**
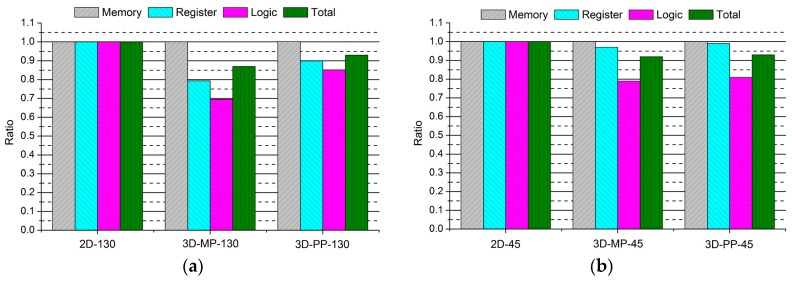
Normalized power comparisons of 2D and 3D ICs: (**a**) 130-nm process technology and (**b**) 45-nm process technology.

**Figure 14:**
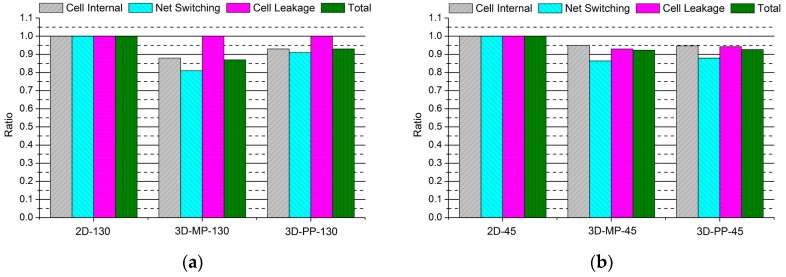
Normalized power comparisons of 2D and 3D ICs: (**a**) 130-nm process technology and (**b**) 45-nm process technology.

To study the source of the power reduction in 3D ICs, we performed various power analyses. First, as shown in Figure 13 and Figure 14, we observe that most power savings are achieved in the combinational logic and net switching power, indicating that the reduced number of buffers in the interconnection plays an important role in reducing the power consumption of 3D IC designs. In addition, we also observe that in contrast to the 3D ICs designed in 130-nm technology, which have negligible cell leakage power, the cell leakage power of the 45-nm 3D IC is as much as 7% less than that of the 2D IC. This is mainly because the number of buffers of the 45-nm process technology-based 3D ICs (3D-MP-45 and 3D-PP-45) are 30% and 35% less than that of 2D IC (2D-45), which results from vertical stacking and the reduced wire length. This result also indicates that as the process technology scales down, cell leakage power increases power consumption mainly because leakage power in 130-nm technology is negligible, but the proportion of leakage power in 45-nm technology is higher than it is in 130-nm technology.

Table 8 and Table 9 show how power consumption breaks down to cell internal, net switching, and cell leakage across power groups. From the tables, we observe that memory macros consume around 38% and 32% of the power in 130-nm and 45-nm process technologies, respectively, because of the memory-intensive nature of the stereo matching processor. In addition, we observe that the clock network consumes a relatively large portion of total power because of its high switching activity. In the case of a 3D IC designed in 130-nm technology, for example, the clock network consumes over 30% of power even though the lengths of wires and the number of buffers of the clock tree comprise only around 4% of all wire length and 5% of all buffers. Comparing 130-nm 3D ICs and 45-nm 3D ICs, we also observe that as the technology scales down, the proportion of switching power increases while the proportion of internal power decreases, indicating that as the technology scales down, switching power leads to greater power savings.

### 4.3. Impact of Switching Activity

**Figure 15:**
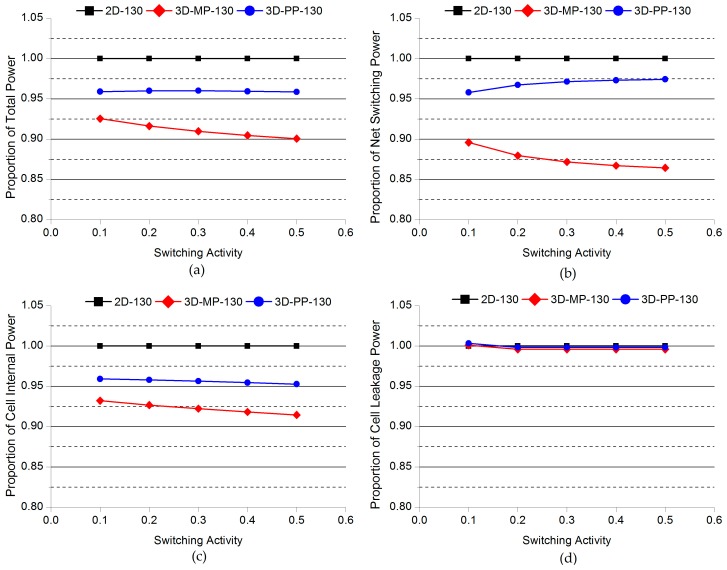
Comparisons of the normalized power of 2D and 3D ICs as a function of switching activity: (**a**) Total power; (**b**) net switching power; (**c**) cell internal power; (**d**) cell leakage power. Note that the power consumption of 2D-130 actually increases as the switching activity increases.

**Figure 16:**
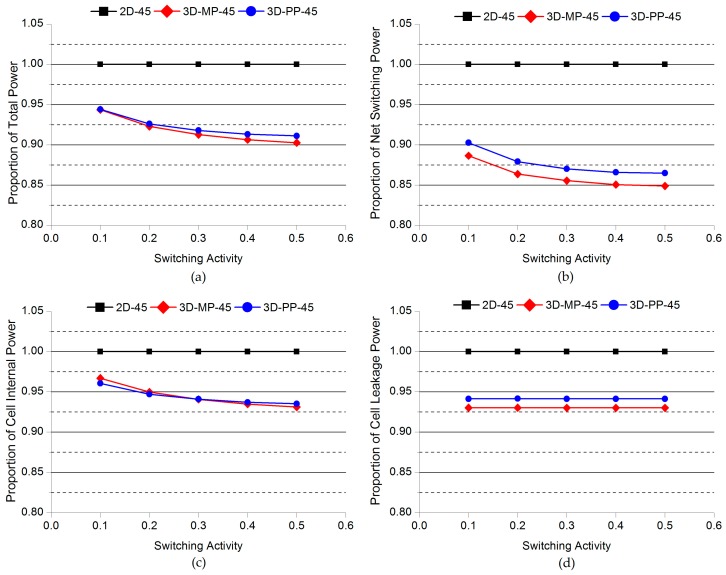
Comparisons of the normalized power of 2D and 3D ICs as a function of switching activity: (**a**) Total power; (**b**) net switching power; (**c**) cell internal power; (**d**) cell leakage power. Note that the power consumption of 2D-45 actually increases as the switching activity increases.

To investigate the impact of switching activity on the power benefits in 3D ICs, we performed a detailed power analysis while varying the switching activity. We specified the target static probability values of the switching activity on the primary inputs, which varied from 0.1 to 0.5 and used PrimeTime to propagate the switching activity to the rest of the circuits. The varying switching activity is therefore represented as the various workloads of stereo matching processors. Figure 15 and Figure 16 present comparisons of the normalized power of 2D and 3D ICs as a function of the switching activity. First of all, we observe that the overall power savings of 3D-MP-130, 3D-MP-45, and 3D-PP-45 over their 2D IC counterparts increase as the switching activity increases. This increase is due to the reduction in power mainly from the shortened wire lengths of the 3D ICs. This finding indicates that as switching activity increases, the power benefits of 3D ICs become more significant, but in the case of 3D-PP-130, power savings over 2D-130 do not change. After all, neither the lengths of the clock-tree wires nor the number of clock buffers of the 3D-PP-130 decrease, confirming that as the switching activity increases, the clock-tree network becomes more important for power savings.

### 4.4. Comparisons of the Results with the Related Studies

We compared the reduction in power and wire length with the related studies, summarized in Table 1. However, because of the different types of case studies and goals, only a few studies compared the 3D IC with its 2D IC counterpart. Table 10 summarizes the comparisons of the power and wire length reduction with the related studies. Ouyang et al. focused on delay reduction in 3D-stacked arithmetic units [7]. Kim et al. [10], Zhang et al. [11], and Saito et al. [12] focused on the feasibility of 3D-stacked IC designs. Thorolfsson et al. [8], Neela et al. [9], Franzon et al. [13] and Oh et al. [14] compared 3D ICs with their 2D IC counterparts. Thorolfsson et al. [8] and Franzon et al. [13] presented a 3D-stacked FFT processor composed of memory on logic and compared it with its 2D IC counterpart. They achieved 4.4% and 56.9% reductions in power and wire length using MIT Lincoln Labs’ manufacturing process. Neela et al. implemented a 3D-stacked single precision floating-point unit. However, it consumes more power than that of its 2D IC counterpart mainly because the increased wire length for the routing signal to micro bumps [9]. Oh et al. [14] demonstrated a 3D-stacked ternary content-addressable memory and achieved 21.5% reduction in power.

## 5. Conclusions

We described the benefits of TSV-based 3D stacking and the impact of device technology scaling on the performance of the stereo matching processor. We also presented comprehensive comparisons of 2D and 3D-stacked stereo matching processors designed in 130-nm and 45-nm technologies. In addition, we presented the overall RTL-to-GDSII design flow and described the power and timing analysis flow for a TSV-based 3D IC design. From the experimental results, we observed that the reduced power consumption of our 3D-stacked stereo matching processors is mainly due to shortened wire lengths and reduced buffer use resulting from 3D stacking. In the case of the 3D ICs designed with 130-nm process technology, our pipeline-level method reduces total power by 7% and our macro-level method by 13%. Likewise, in the case of 3D ICs designed with 45-nm process technology, our pipeline-level method achieved 7% and our macro-level partitioning method 8% savings.

This paper demonstrated significant power reductions with our 3D-stacked stereo matching processors designed in either the 130-nm or the 45-nm process technologies. From the experimental results, we showed that as the switching activity increases, the clock-tree network and buffers of 3D ICs consume less power. In addition, we showed that the proposed pipeline-level partitioning method minimizes TSV usage while balancing the area of each tier. The reduction in the number of signal TSVs, however, does not always lead to an optimal design with regard to total wire length and power reduction. Therefore, to fully exploit the benefits of vertical stacking, designers should optimally place TSVs in physical layouts.

## Figures and Tables

**Table 1:** Related studies on 3D-stacked IC design.

Authors	3D IC Design	Process Technology	Key Features
Ouyang et al. [7]	Arithmetic units with three logic tiers	Massachusetts Institute of Technology (MIT) Lincoln Lab’s 180 nm	11.0% ~ 46.1% reduction in power
Thorolfsson et al. [8]	FFT processor with two logic tiers and one static random access memory (SRAM) tier	MIT Lincoln Lab’s 180 nm	56.9% reduction in wire length, 4.4% reduction in logic power
Neela et al. [9]	Single precision floating-point unit with two logic tiers	GlobalFoundries 130 nm	41.5% reduction in footprint, 3% increase in frequency
Kim et al. [10]	64 processors with one logic tier and one SRAM tier	GlobalFoundries 130 nm	63.8 GB/s memory bandwidth, power consumption up to 4.0 W
Zhang et al. [11]	Syntem-on-Chip with two logic tiers and three DRAM tiers	GlobalFoundries 130 nm	12.57 mW power consumption, 8.5 GB/s bandwidth
Saito et al. [12]	Dynamic-reconfigurable memory with one logic tier and one SRAM tier	90-nm process technology	63% reduction in area and 43% reduction in latency
Franzon et al. [13]	Digital signal processor with two logic tiers and one SRAM tier	MIT Lincoln Lab’s 180 nm	25% reduction in total power (logic and memory)
Oh et al. [14]	Ternary content-addressable memory with three tiers	MIT Lincoln Lab’s 180 nm	21% reduction in total power

**Table 2:** Main features of our stereo matching processors designed in 130- and 45-nm technologies.

Feature Type	130-nm Process Technology	45-nm Process Technology
Maximum frequency	312 MHz	556 MHz
Image size (pixel)	752 × 480	752 × 480
Window size (pixel)	15 × 15	15 × 15
Disparity range (pixel)	64	64
Maximum frame rate	108 frames/s	192 frames/s
Maximum bandwidth	12.8 GB/s	22.8 GB/s

**Table 3:** Summary of synthesis results.

Process Technology	Area (μm × μm)	Clock Period	# of SRAM	SRAM Capacity
130-nm	2,977,542	3.2 ns	44	44 × 752 bytes = 31.3 kB
45-nm	451,797	1.8 ns	44	44 × 752 bytes = 31.3 kB

**Table 4:** TSV usage comparisons.

Partition Method	Type of Signal	# of TSVs
Macro-level Partition (MP)	SRAM control signals	29
	SRAM address signals	20
	SRAM data signlas	376
	Total Number of TSVs	425
Pipeline-level Partition (PP)	Logic signals between pipeline stages	56
	SRAM control signals	41
	SRAM address signals	20
	SRAM data signlas	104
	Total Number of TSVs	221

**Table 5:** Cell area of each pipeline stage of the stereo matching processors designed in 130- and 45-nm technologies.

# of Pipeline Stage	Name	130-nm Process Technology		45-nm Process Technology	
		Cell Area (μm × μm)	%	Cell Area (μm × μm)	%
1	32 SRAM macros	1,294,065	43.5	194,109	43.0
2	Hamming weight	255,783	8.6	42,660	9.4
3	Hamming distance	459,072	15.4	68,576	15.2
4	12 SRAM macros	485,274	16.3	72,791	16.1
5	Median filter	241,725	8.1	32,831	7.3
6	Disparity diffusion	241,623	8.1	40,831	9.0

**Table 6:** Overall layout results of 2D and 3D ICs designed in 130-nm process technology.

	2D-130	3D-MP-130		3D-PP-130	
		Top Tier	Bottom Tier	Top Tier	Bottom Tier
Clock period (ns)	3.2	3.2	3.2	3.2	3.2
Footprint (μm × μm)	2350 × 2350	2350 × 1350	2350 × 1350	2350 × 1350	2350 × 1350
# of gates	97,630	87,245	205	50,113	39,528
Total wire lengths (μm)	5,488,514	4,507,365	220,485	2,662,651	2,582,875
Clock net wire lengths	231,232	184,683	18,293	119,704	95,636
Total # of buffers	18,968	15,242	161	8575	7051
# of clock tree buffers	871	740	61	508	399
Power (mW)	1006.31	871.04		931.30	

**Table 7:** Overall layout results of 2D and 3D ICs designed in 45-nm process technology.

	2D-45	3D-MP-45		3D-PP-45	
		Top Tier	Bottom Tier	Top Tier	Bottom Tier
Clock period (ns)	1.8	1.8	1.8	1.8	1.8
Footprint (μm × μm)	830 × 830	830 × 485	830 × 485	830 × 485	830 × 485
# of gates	123,659	101,640	165	58,745	40,859
Total wire lengths (μm)	1,918,625	1,629,674	75,494	988,637	881,191
Clock net wire lengths	86,758	75,249	5950	47,981	38,455
Total # of buffers	34,520	24,198	121	12,704	9863
# of clock tree buffers	1451	1415	52	909	692
Power (mW)	273.79	252.56		253.44	

**Table 8:** Detailed power comparisons of 2D and 3D ICs designed in 130-nm technology.

Design Type	Power Group	Cell Internal (mW)	Net Switching (mW)	Cell Leakage (mW)	Total (mW)	Percentage (%)
2D-130	Memory	353.70	1.33	0.08	355.11	35.29
	Clock Network	246.10	71.0	0.00	317.10	31.51
	Register	41.0	14.70	0.00	55.70	5.54
	Combinational Logic	135.80	142.60	0.00	278.40	27.67
	Total	776.60	229.63	0.08	1006.31	100.0
	Percentage (%)	77.17	22.82	0.01	100.00	n/a
3D-MP-130	Memory	353.80	1.46	0.08	355.34	40.79
	Clock Network	209.70	68.20	0.00	277.90	31.90
	Register	31.80	12.40	0.00	44.20	5.07
	Combinational Logic	89.80	103.80	0.00	193.60	22.23
	Total	685.10	185.86	0.08	871.04	100.00
	Percentage (%)	78.65	21.34	0.01	100.00	n/a
3D-PP-130	Memory	353.70	2.02	0.08	355.80	38.20
	Clock Network	219.50	68.80	0.00	288.30	30.96
	Register	36.40	13.70	0.00	50.10	5.38
	Combinational Logic	113.80	123.30	0.00	237.10	25.46
	Total	723.40	207.82	0.08	931.30	100.00
	Percentage (%)	77.68	22.32	0.01	100.00	n/a

**Table 9:** Detailed power comparisons of 2D and 3D ICs designed in 45-nm technology.

Design Type	Power Group	Cell Internal (mW)	Net Switching (mW)	Cell Leakage (mW)	Total (mW)	Percentage (%)
2D-45	Memory	79.20	0.39	5.05	84.64	30.9%
	Clock Network	37.30	28.10	0.13	65.53	23.9%
	Register	15.20	5.44	0.72	21.35	7.8%
	Combinational Logic	49.90	50.40	1.97	102.27	37.4%
	Total	181.60	84.32	7.87	273.79	1.00
	Percentage (%)	66.33	30.80	2.87	100.00	n/a
3D-MP-45	Memory	79.2	0.4548	5.051	84.71	33.5%
	Clock Network	38.4	28	0.1299	66.53	26.3%
	Register	15.2	4.687	0.7167	20.60	8.2%
	Combinational Logic	39.6	39.7	1.419	80.72	32.0%
	Total	172.40	72.84	7.32	252.56	100
	Percentage (%)	68.26	28.84	2.90	100.00	n/a
3D-PP-45	Memory	79.2	0.6416	5.051	84.89	33.5%
	Clock Network	36.5	28.4	0.1349	65.03	25.7%
	Register	15.2	5.296	0.7156	21.21	8.4%
	Combinational Logic	41.1	39.7	1.505	82.31	32.5%
	Total (mW)	172.00	74.04	7.41	253.44	100
	Percentage (%)	67.87	29.21	2.92	100.00	n/a

**Table 10:** Comparisons of the power and wire length reductions in this study with those in related studies.

			Power (mW)			Wire Length (m)		
			2D IC	3D IC	∆ (%)	2D IC	3D IC	∆ (%)
Proposed	2D-130	3D-MP-130	1006.3	871	−13.4%	5.489	4.728	−13.9%
		3D-PP-130		931.3	−7.5%		5.246	−4.4%
	2D-45	3D-MP-45	273.8	252.6	−7.7%	1.919	1.705	−11.1%
		3D-PP-45		253.4	−7.5%		1.870	−2.5%
Related Studies	Ouyang et al. [7]		n/a	n/a	n/a	n/a	n/a	n/a
	Thorolfsson et al. [8]		340.0	324.9	−4.4%	19.107	8.238	−56.9%
	Neela et al. [9]		9.95	10.72	7.7%	10.37	10.96	5.7%
	Kim et al. [10]		n/a	4032.0	n/a	n/a	n/a	n/a
	Zhang et al. [11]		n/a	12.57	n/a	n/a	n/a	n/a
	Saito et al. [12]		n/a	120.0	n/a	n/a	n/a	n/a
	Franzon et al. [13]		340.0	324.9	−4.4%	19.107	8.238	−56.9%
	Oh et al. [14]		0.042	0.033	−21.5%	n/a	n/a	n/a
